# Skin mucosome activity as an indicator of *Batrachochytrium salamandrivorans* susceptibility in salamanders

**DOI:** 10.1371/journal.pone.0199295

**Published:** 2018-07-18

**Authors:** Hannah Keely Smith, Frank Pasmans, Maarten Dhaenens, Dieter Deforce, Dries Bonte, Kris Verheyen, Luc Lens, An Martel

**Affiliations:** 1 Wildlife Health Ghent, Department of Pathology, Bacteriology & Avian Diseases, Ghent University, Salisburylaan, Merelbeke, Belgium; 2 Laboratory for Pharmaceutical Biotechnology, Ghent University, Faculty of Pharmaceutical Science, Ottergemsesteenweg, Ghent, Belgium; 3 Terrestrial Ecology Unit (TEREC), Department of Biology, Ghent University, K. L. Ledeganckstraat, Ghent, Belgium; 4 Forest & Nature Lab, Department of Environment, Ghent University, Geraardsberge Steenweg, Gontrode, Belgium; Universitat Trier, GERMANY

## Abstract

Recently emerged fungal diseases, *Batrachochytrium dendrobatidis* (*Bd*) and *Batrachochytrium salamandrivorans* (*Bsal*) are an increasing threat to amphibians worldwide. In Europe, the threat of *Bsal* to salamander populations is demonstrated by the rapid decline of fire salamander populations in Germany, the Netherlands and Belgium. Although most European urodelans are susceptible to infection in infection trials, recent evidence suggests marked interspecific differences in the course of infection, with potentially far reaching implications for salamander conservation. As a salamander’s skin is the first line of defense against such pathogens, interspecific differences in innate immune function of the skin may explain differential susceptibility. Here we investigate if compounds present on a salamander’s skin can kill *Bsal* spores and if there is variation among species. We used a non-invasive assay to compare killing ability of salamander mucosomes of four different species (captive and wild *Salamandra salamandra* and captive *Ichtyosaura alpestris*, *Cynops pyrrhogaster* and *Lissotriton helveticus*) by exposing *Bsal* zoospores to salamander mucosomes and determining spore survival. In all samples, zoospores were killed when exposed to mucosomes. Moreover, we saw a significant variation in this *Bsal* killing ability of mucosomes between different salamander host species. Our results indicate that mucosomes of salamanders might provide crucial skin protection against *Bsal*, and could explain why some species are more susceptible than others. This study represents a step towards better understanding host species variation in innate immune function and disease susceptibility in amphibians.

## Introduction

Wildlife diseases are an increasing threat to biodiversity [1,2]. Over the last two decades amphibian populations have declined due to recently emerged diseases [3–5]. Among them, fungal pathogens, such as *Batrachochytrium dendrobatidis* (hereafter *Bd*) and *Batrachochytrium salamandrivorans* (hereafter *Bsal*), etiological agents of chytridiomycosis have been wreaking havoc on amphibian populations worldwide [6–12]. In Europe, *Bsal* emerged suddenly and severely, causing a population of fire salamanders to nearly collapse in the Netherlands, with less than 0.1% of the population remaining [13,14]. Similar population collapses occurred in Belgium [15] and have been found in Germany [16]. Probably originating in Asia [17,18] this pathogen may have a quick and devastating effect on most salamander species of Europe. The risk of further spread globally has caused proactive responses in the form of taskforces to emerge and trade restrictions to be implemented in the USA, Canada and Switzerland [19,20].

*Batrachochytrium salamandrivorans* differs from its sister species (*Bd*) in that the disease is limited to urodelans but, like *Bd*, the effect it has on its host differs greatly among but also within species [15,17,21]. Previous infection trial studies observed a range of host susceptibility, from highly susceptible (ie. fire salamanders *Salamandra salamandra)* to moderately susceptible (ie. alpine newt *Ichtyosaura alpestris* and Japanese fire belly newt *Cynops pyrrhogaster*) to resistant (ie. palmate newt *Lissotrition helveticus*) [15]. The differences seen in disease susceptibility could be partly the result of immune defenses, as these are known to vary among individuals and species [22]. In addition to host immune factors, susceptibility of amphibians to *Bd* has been attributed to many different factors including skin microbiota [23–25], genetic makeup of populations [26] and environmental factors [27,28].

Given the increasing threat of new pathogens to amphibians, understanding host immune function and susceptibility is increasingly important [22]. In amphibians, the first line of defense against chytrid pathogens is their skin [29]. As amphibians use their skin for a multitude of key physiological functions (e.g. chemical defenses, thermo- and osmoregulation) their skin is a particularly important organ, vulnerable to invading pathogens [30]. *Bd* and *Bsal* target and invade the amphibian’s skin, thereby interfering with the skin’s vital functions [31,32]. Therefore, understanding innate immune factors present on the skin of amphibians, and how this immune function varies between individuals and species is important.

Amphibian skin generally has two types of specialized secretory glands which help protect the skin. Firstly, granular glands produce defensive secretions that contain a cocktail of different bioactive molecules (i.e. alkaloids, TTX toxins, steroids, amines, antibodies, lysozymes and antimicrobial peptides) [29, 33–39]. The exact molecules vary widely among amphibians and are used for predator deterrence and host immunity. In many amphibian species, these glands are concentrated in large conspicuous structures [40] that release their poisonous content upon irritation, mechanical pressure, stress or adrenaline stimulation [41]. Besides releasing bioactive components when stimulated, studies have shown that the granular glands of non-stressed frogs also release such components into the mucous layer at low levels [42]. Secondly, mucous glands produce a mixture of mucin glycoproteins that make up the main component of the skin mucosal layer and can behave as a physical barrier to pathogenesis [29]. Additionally, the skin and mucous layer have been shown to harbor a community of microbiota (and their metabolites), believed to influence growth of pathogens [43–45]. Together the mixture of mucin glycoproteins, granular gland secretions [46] and the skin microbiota make up the mucosome [46,47].

Research on amphibian skin immune function focuses largely on frog species and the effect of AMPs which have been shown to reduce infection loads of *Bd*, in some cases allowing the animal to clear the infection [29,42,48–51]. The long history of research in skin secretions of salamanders has revealed a plethora of defensive compounds [52–54]. Within Salamandridae, several species produce alkaloids, namely samandarine and samandarone [34,37,55,56] and TTX toxins in their secretions [36,38,39]. However, comparatively few studies have shown the effect of salamander skin secretions against *Bd*, with a few exceptions [57–60]. Here we investigate whether salamander mucosomes and skin secretions can affect *Bsal* and/or *Bd* spore viability using the vulnerable fire salamander as our main model. Additionally, we examined whether the innate immune function of the salamander’s mucosome differs among host species in its killing ability of *Bsal*.

## Materials & methods

### Study animals and husbandry

To determine the extent to which fire salamander skin secretions and fire salamander, alpine newt, palmate newt and Japanese fire belly newt mucosomes are capable of killing *Bsal* zoospores, we sampled mucosomes from captive born and raised animals. All animals used were adults and randomly selected from their terraria or tanks. Fire salamanders, alpine newts and palmate newts were selected as they are all European species co-occurring in *Bsal* infected forests and have differing susceptibilities during infection trails. The Japanese fire belly newt was included as an Asian species suspected of being a *Bsal* carrier. Fire salamanders were housed in large mesocosms of 2.5 m by 1.25 m in groups of 10–15 animals with moist soil and dry leaves and clay tiles for shelter and kept at 8°C—15°C. Palmate newts, alpine newts and Japanese fire belly newts were housed separately in large glass tanks of 40 cm by 60 cm in groups of 10, on moist soil and dry leaves with clay tiles for shelter and kept between 15°C—20°C. For all animals, appropriate food (crickets, worms or bloodworms) was provided *ad libitum*. In addition, mucosomes of fifteen wild fire salamander were collected in November 2016 from Makegem- Harentbeek forest East Flanders, Belgium. A 54.24 ha private forest with 10 ponds (latitude 50.945331, longitude 3.714886). Animal experiments were conducted according to biosecurity and ethical guidelines set forth by the ethical committee of the Faculty of Veterinary Medicine, Ghent University. Ethical permission was not required under Belgian Legislation (Law 14 August 1986 related to the protection of animals). Permits for sampling of wild fire salamanders were granted by Agentschap voor Natuur en Bos of East Flanders in Belgium, license number ANB/BL/FF-V15-00015.

### Experimental procedure

Mucosome bathing was performed on all salamanders and newts as described in Woodhams et al. 2014 [46]. Each amphibian was rinsed prior to bathing, and its surface area was calculated according to Spight et al. 1968 (surface area in cm^2^ = 8.42*(mass in g)^(0.694) and divide surface area by 4 to determine quantity of water to add)[61]. Animals were bathed in HPLC water for 1 hour and the wash solution was collected and frozen at -20°C until further analysis.

Fire salamander skin secretions were collected by massaging the granular glands with a microbiological inoculation loop rubbed over the dorsal tail. Collected skin secretions where weighed to the nearest 0.1 mg. Varying amount of skin secretions where collected per individual, ranging from 5 mg to 20 mg. To create a skin secretions suspension HPLC water was added to the sample at a ratio of 10 μl of water to 1 mg of secretions. The samples were then sonicated in a sonication bath (Branson 2510 ultrasonication bath) for 15 mins (120V, 60Hz). Samples were frozen at -20°C until analysis.

We performed experiments in which we exposed *Bd* JEL 423 and *Bsal* AMFP 13/01 zoospores (10^6^ per mL) to the mucosome bathing solution or skin secretions (Palmate newt/Japanese fire belly newt n = 6, captive fire salamander/alpine newt n = 8, wild fire salamander n = 15). *Bd* JEL 423 and *Bsal* AMFP 13/01 cultures were grown in flasks in TGhL (tryptone, gelatin hydrolysate and lactose) broth at 20°C and 15°C respectively. During sporulation, zoospores were harvested and filtered (filter size 10 μm) to remove sporangia. Spore concentration was determined by haemocytometer count using lugol solution (Sigma) to stain zoospores. Zoospore suspensions of 1 x 10^6^ mL were used for all assays.

We used trypan blue staining to determine viability of *Bd* and *Bsal* spores after exposure to amphibian mucosomes or glandular gland skin secretions. The protocol was adapted from McMahon and Rohr 2014 [62].

The *Bd* or *Bsal* survival assays were performed by adding zoospore suspension to mucosome and skin secretion solution and incubating for 60 minutes. The number of viable *Bd* or *Bsal* zoospores in the zoospore suspension was determined before inoculation. HPLC water (treated as wash solution without the salamander) was used to provide a background survivability of *Bd* or *Bsal* spores, heat-killed spores were used as positive control. After incubation, the numbers of remaining viable zoospores were calculated using trypan blue dye (0.4% trypan blue in phosphate buffered saline). Viable spores were counted using a hemocytometer and compound microscope. All tests were performed blind and repeated in triplicate. Secretion samples were completely sterilized by one hour of UV radiation to eliminate possible effects of live bacteria in our experiments. Mucosome solutions were sterilized using 0.2 μm filters.

We calculate the killing ability of each animal’s skin defenses by comparing the (mean) viable chytrid spores after inoculation with mucosome (or secretions) versus the negative control (viable spores in water) to calculate the percent viable spores.

### Skin defenses components

To investigate the components responsible for the observed *Bsal* killing ability, we collected skin secretions of fire salamanders and mucosomes of alpine newts as described above, the latter were used as their mucosomes exhibited marked zoospore killing activity.

In order to investigate whether the bioactive compounds are large (high molecular weight) or small (low molecular weight) molecules, we separated components based on size using dialysis with different membrane pore sizes. Dialysis membranes (Spectra/Por Micro Float-A-Lyzer and Specta/Por 6 Dialysis Tubing), size 50 kD were used per the manufacturer’s instructions. Samples were loaded into the filter and spun for 16 hours at 4°C to remove smaller particles. The samples were then used to run the *Bsal* spore viability assay.

To test whether the active components could be proteins or peptides we denatured all proteins and peptides present in the secretions and mucosomes using Endoproteinase GluC (*Staphylocococcus aureus* Protease V8, New England BioLabs), according to the manufacturer’s instructions. Digestion of proteins/peptides was achieved by adding GluC Reaction Buffer to sample and Endoproteinase GluC for 16 hours at 37°C. The samples were then used to run the *Bsal* spore viability assay.

To examine the proteins present in fire salamander skin secretions (diluted 1:100) we performed SDS-PAGE (Sodium dodecyl sulfate polyacrylamide gel electrophoresis) Mini-Protean 3 Cell gel (Bio-Rad) with Brilliant blue G-Colloidal concentrate staining used by manufactures specifications (Sigma).

### Mass spectrometry

Further examination of fire salamander skin secretion proteins was done by in-gel digestion followed by high resolution LCMSMS analysis (Synapt G2Si, Waters) using HDDDA acquisition. Data was searched using an in-house Mascot Server against a Uniprot Amphibia library.

### Statistical analysis

All analyses were performed in SPSS version 24, and all data were checked for normality and equality of variance among groups prior to statistical analysis. A Student’s t-test (normal distribution) or Mann Whitney U test (non-normal distribution) was performed to examine the difference in viable *Bd* and *Bsal* spores after inoculation with the fire salamander mucosome and secretion. To test whether the mucosome killing-ability varies among species, we performed a one-way analysis of variance (ANOVA) followed by a Tukey (equal variances assumed) or Games-Howell (equal variances not assumed) post hoc test.

## Results

### Skin defenses function

**Fig 1:**
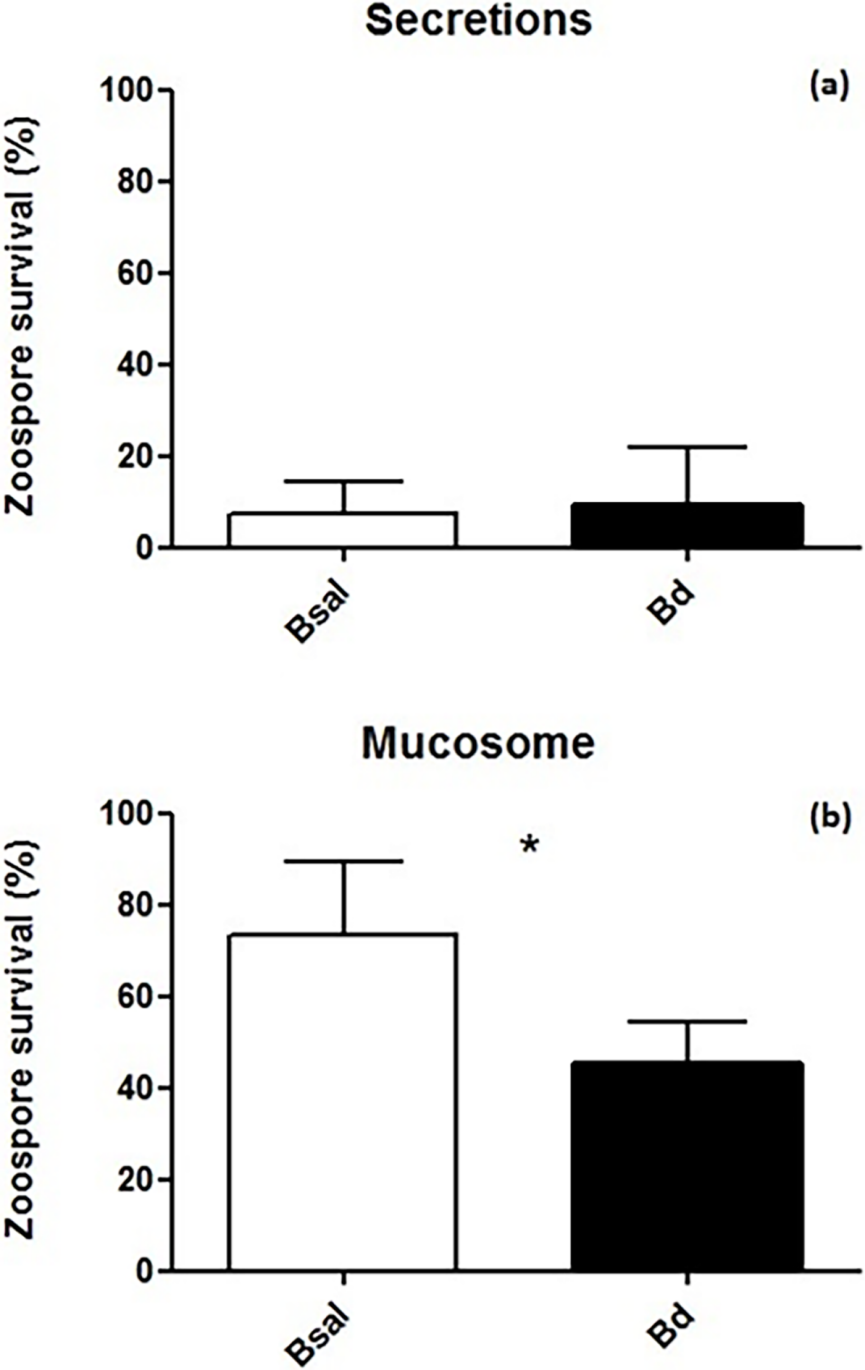
a) Fire salamander secretion killing activity versus *Bd* and *Bsal* zoospores. b) Fire salamander mucosome killing activity versus *Bd* and *Bsal* zoospores. Secretions (diluted 1:10) and mucosome of fire salamander (n = 8). Shown are the mean +/- SD of the percentage of viable zoospores observed after 60 minutes of incubation (compared with viable spores exposed to water). Asterisks indicate a significant difference at p < 0.05.

Fire salamander skin secretions were shown to drastically reduce viable spores of *Bsal* and *Bd*, resulting in more than 80% spore mortality after one hour in both chytrid species (Fig 1A). *Bsal* and *Bd* spore mortality were not significantly different with 92.5% and 90.5%, respectively (*U* = 32, *p* = 1.00). The mucosome of fire salamanders killed a substantial amount of chytrid spores (Fig 1B), however, reduction in viable *Bd* spores (54.5%) was significantly greater than *Bsal* spores (26.5%) (*t* = 4.4, df = 11 *p* = 0.001). The killing ability of salamander secretions on both chytrid species was markedly higher than salamander mucosome solutions (compare Fig 1A to 1B).

**Fig 2:**
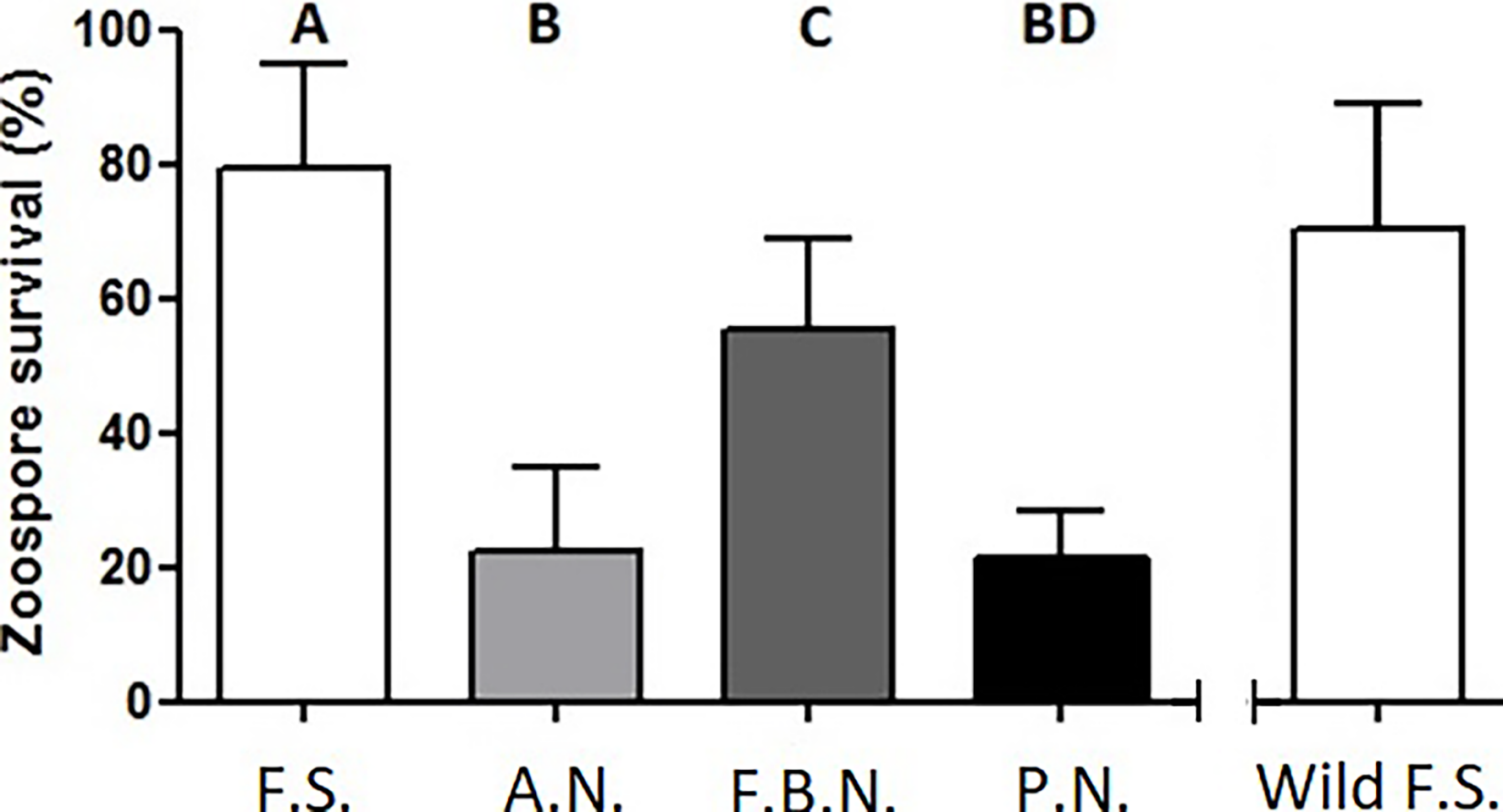
*Bsal* zoospore survival after exposure to different species mucosomes. F.S. = fire salamander (n = 8), A.N. = alpine newt (n = 8), F.B.N. = Japanese fire belly newt (n = 6), P.N. = palmate newt (n = 6). Wild F.S. are depicted for comparison but originated from a separate experiment (n = 15). Shown are the mean +/- SD of the percentage of viable zoospores observed after 60 minutes of incubation (compared with control, viable spores in water). F.S. vs A.N. (p < 0.001), F.S. vs F.B.N. (p = 0.011), F.S. vs P.N. (p < 0.001), A.N. vs F. B. N. (p < 0.001), A.N. vs P. N. (p = 0.998).

*Bsal* spore mortality varied significantly between the mucosomes of the four different salamander species (ANOVA: *F* = 35.032, df = 3, 24, *p* < 0.001) (Fig 2). The only two mucosomes that did not differ significantly in their *Bsal* killing activity were those of the alpine newts and palmate newt (*p* = 0.998). These two species’ mucosomes killed a significant portion of *Bsal* spores, with 77.6% and 78.9% spore mortality respectively. Captive fire salamander mucosomes killed the fewest spores with an average 20.7% spore mortality, while fire belly newts killed a higher percentage of spores (average of 45% spore mortality). These results correlate with the known susceptibility of these species when infected with *Bsal* during clinical trials [15,17]. To check if mucosome activity of captive fire salamanders is similar to that of wild fire salamanders, we tested the mucosome activity of 15 wild fire salamanders against *Bsal* zoospores. This resulted in an average *Bsal* spore mortality of 28.8%, which is comparable to captive fire salamanders mucosomes (Fig 2). Wild fire salamanders displayed a slightly larger variation in *Bsal* killing ability, ranging between 0 to 63 percent spore mortality, compared to 2 to 40 percent in captive animals.

### Protein analysis

**Fig 3:**
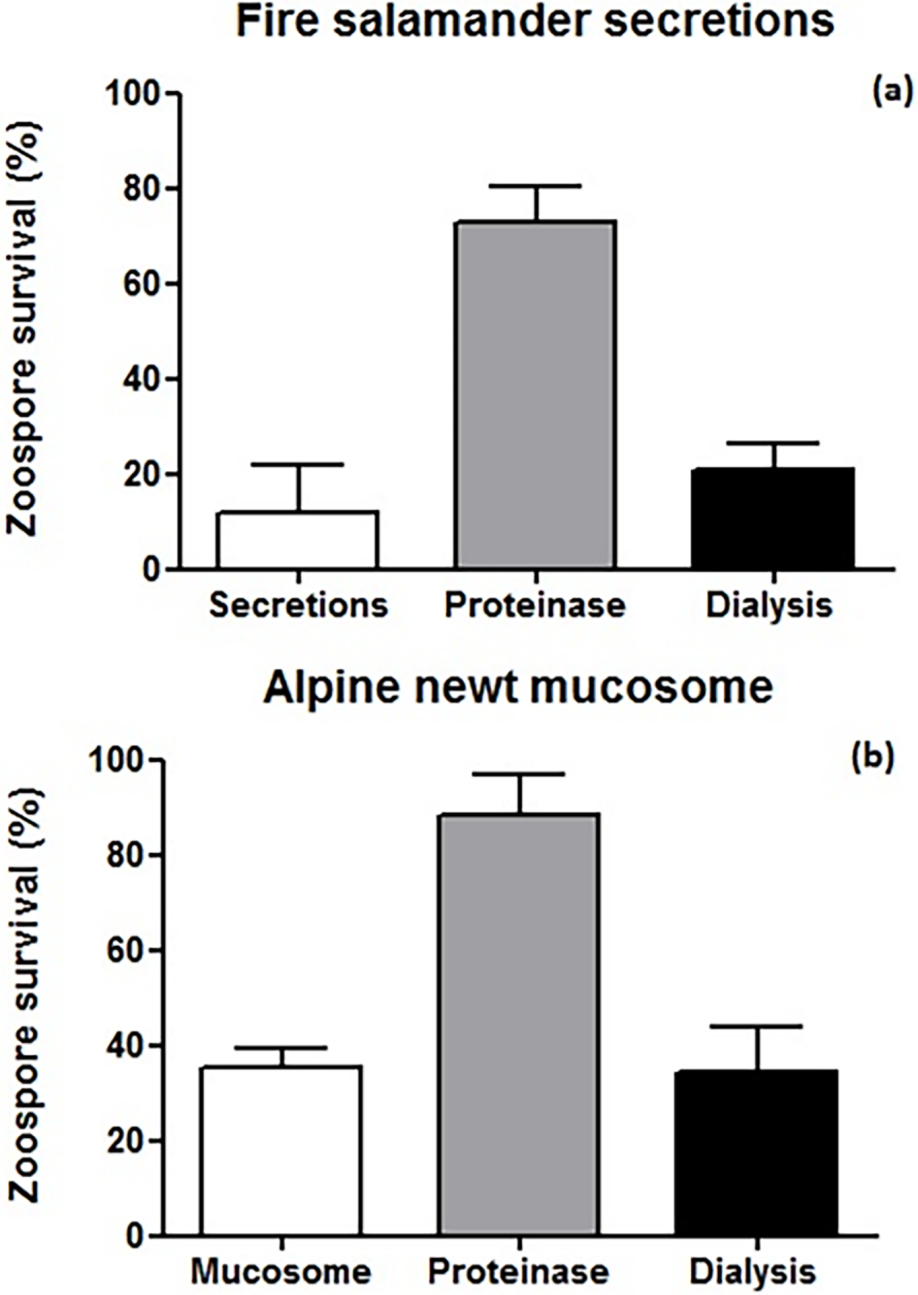
a) *Bsal* zoospore survival in the presence of treated and untreated fire salamander skin secretions. Pooled secretions (diluted 1:100, n = 3) b) *Bsal* zoospore survival with treated and untreated alpine newt mucosomes. Pooled mucosomes (n = 13). Shown are the mean +/- SD of the percentage of viable zoospores observed after 60 minutes of incubation (compared with control, viable spores in water).

Protein denaturation of fire salamander skin secretions resulted in a decrease in *Bsal* killing-ability, with a major decrease in spore mortality of 39.1% compared to the control treatment (Fig 3A). When samples were filtered with a 50 kD dialysis membrane (to remove smaller molecules), we didn’t observe an apparent reduction in the killing activity of the secretions (Fig 3A). Similar effects were observed when testing the killing activity of alpine newt mucosomes after protein denaturation and dialysis (compare Fig 3A to 3B).

The banding pattern on SDS-PAGE gel used to examine proteins from the secretions, ranging in size from proteins at ~16 kD to ~250 kD. Prominent bands were seen at ~16 kD, ~70 kD, ~80 kD and ~250 kD. Proteins and peptides of a mass below 16 kD were not detected (S1 Fig).

Analysis of protein bands by LCMSMS resulted in tentative identification as mostly structural/cellular proteins. However, annotation results and further identification of candidate immune/defence proteins were inconclusive due to poor proteome coverage of our target species.

## Discussion

As the surface of the skin represents the first contact between the amphibian host and its chytrid pathogen, the innate immune function of the skin is a key first step influencing subsequent disease dynamics. We found that components on urodelan skin can kill zoospores of both *Bd* and *Bsal*. Moreover, *Bsal* killing activity of the mucosome of different salamander’s species reflect their known susceptibility.

Focusing on skin defenses of fire salamanders, skin secretions were found to be surprisingly effective in killing both *Bd* and *Bsal* spores with ten to hundred-fold dilutions of skin secretion samples killing the great majority of *Bsal* and *Bd* spores. On the contrary, mucosomes of both captive and wild fire salamanders are less effective at killing *Bsal* spores. This apparent difference between mucosomes and granular gland secretions could be due to the fact that active components originate from the secretions and are still present in the mucosome, but at a more diluted concentration. High quantities of secretions are only produced during irritation, mechanical pressure, stress or adrenaline stimulation [41,42]. Interestingly, fire salamander mucosome activity against *Bd* and *Bsal* reflects the differences in their susceptibility to these two chytrid species (Fig 1). Fire salamander are highly susceptible to *Bsal*, with 100% lethality of fire salamanders during infection trials [17], and field reports show that its effects in the wild are similarly lethal [14,15]. Conversely, *Bd* does not kill fire salamanders in clinical trials, but published data on this is lacking due to the difficulty in establishing *Bd* infection in the host. Furthermore, the only report of *Bd* having a lethal effect on fire salamanders originates from the Peñalara Natural Park, Spain [63].

Palmate newts are apparently resistant to *Bsal* during infection trials [17] and there have been no observations of wild palmate newts being infected even though these newts are found within environments where *Bsal* is present. The mucosome of this species killed three-quarters of *Bsal* spores in one hour. In addition to the highly susceptible fire salamander and the resistant palmate newt we tested two moderately susceptible species, alpine newts and Japanese fire belly newts. The effect of *Bsal* on alpine newts is dose-dependent. During infection trials with a high dose of *Bsal* the animals become sick and die [17], whereas when the dose is lower animals can recover and clear the infection [15]. Although there are some reports of infected alpine newts in the wild, prevalence is low (<10%) and no population declines have been observed [16] even when co-occurring with infected fire salamanders. While other factors could be at play, this suggests that a low infection dose of *Bsal* could possibly be more representative of natural disease dynamics.

We show that the alpine newt mucosome kills larger amount of *Bsal* spores than the fire salamander, suggesting that the mucosomes aid the species in clearing the disease. We see a marked similarity in the mucosome activity of the alpine newt and palmate newt, but not in their susceptibilities. In infection trials *Bsal* has proven to be highly invasive in alpine newt skin but not in the palmate newt [17,64], suggesting that for the palmate newt, the skin itself could be playing a role in its susceptibility, in addition to the mucosome. The Japanese fire belly newt is moderately susceptible to *Bsal*, at high infection doses around half infected animals die and half recover [17]. Here we found that the mucosome kills roughly half of exposed spores. Although it could be assumed that both the fire belly newt and the alpine newt should have similar mucosome killing activity, the statistical discrepancy between the mucosome function of these species can be explained. Firstly, by the large variation seen in alpine newt mucosomes (Fig 2 versus Fig 3B), secondly, although the killing activity of Japanese fire belly newts’ mucosomes might not be as great as those of alpine newts, its susceptibility status could be due to more adaptive immune factors as a result of co-evolution with *Bsal* in Asia [18].

Previous work investigating skin defenses of amphibians against *Bd* has focused largely on frog species; their antimicrobial peptides [29,42,48–51] and their bacterial metabolites [65–69]. There is however, a rapidly growing body of work on skin microbiota of salamanders [70–72]. Furthermore, studies of *Plethodon* salamander skin bacterial metabolites have shown anti-*Bd* activity [73–76]. But a lot of questions remain in regards to salamander skin immune compounds, and their activity against the newly emerged *Bsal*.

Surprisingly, our data indicate that the active anti-chytrid molecules in both the secretions and mucosomes are proteins. Elimination of bacteria from the samples and removal of the low molecular weight fraction (small active molecules like bacterial metabolites) did not affect the ability to kill the spores. This suggests that the main active compounds are not the commonly studied bacterial metabolites, alkaloids or steroidal salamander toxins. Denaturing the protein fraction in both skin secretions and mucosomes drastically reduced the *Bsal* killing ability, which together with the prominent bands on the SDS-PAGE gel suggest the presence of one or several bioactive proteins. Mass spectrometry revealed the presence of proteins but unfortunately, identification of candidate bioactive proteins by LCMSMS mass spectrometry in the skin secretions was unsuccessful due to poor proteomic coverage of our model species. However, several antimicrobial and cytotoxic proteins (in the range of 6 to 72 kDa) like bactericidal proteins, lysozymes, lectins, protease inhibitors and βγ-crystallins have been characterized in the skin secretions of a wide range of frog species [77–80] and analogous compounds may be present in the understudied secretions of urodelans. Future studies using a combination of transcriptomics (e.g. RNAseq of the skin), proteomics and genomics could help identify candidate immune compounds, and thereby broaden our knowledge on salamander innate immunity.

In our case, fire salamander mucosome function mirrors the susceptibility of *Bd* and *Bsal* reported from wild animals and experimental data more accurately than the salamander’s secretions. This is further exemplified by the congruence between the mucosome activity and *Bsal* susceptibility of our other tested salamander species. Although increased secretion of granular gland contents is well known in a state of acute stress (e.g. during a predator threat) [42,52,53], evidence for increased secretion during the initial state of infection remains scarce and inconclusive. It seems logical to study constitutive skin defenses, particularly as the mucosome of amphibians has been shown to contain many factors that can affect chytrid viability. Although the study of skin secretions has proven its immense value for understanding amphibian immunity and skin defenses, our case shows that incorporating mucosome data can result in a more holistic view when studying host susceptibility.

The fact that mucosome activity of captive and wild fire salamanders is highly comparable, indicates that our data obtained from captive animals is biologically relevant, mirroring the conditions found in the wild. Mucosomes as predictors of *Bsal* susceptibility could be used as a crude first step in identifying highly susceptible species or resistant species without the use of infection trials. This method could be of particular importance with endangered species, where sacrificing animals for an infection trail is not possible.

Our study is the first to investigate skin defenses of urodelans against the recently emerged pathogen *Bsal*. We find that skin defenses could play a role in protecting salamanders from this pathogen, which represents an important step towards understanding species variation in disease susceptibly. As the introduction of *Bsal* poses a great risk to native salamander communities, increased knowledge of susceptibility could aid in conservation efforts enabling more focused and effective conservation strategies.

## Supporting information

S1 Fig Fire salamander secretions SDS-PAGE gel. (PDF)

S1 Appendix Tables A–C *Bd* and *Bsal* zoospore assay results. (PDF)
