# Supplementary figures and images for: Skin mucosome activity as an indicator of Batrachochytrium salamandrivorans susceptibility in salamanders
Source: PLoS One. 2018 Jul 18;13(7):e0199295. doi: 10.1371/journal.pone.0199295 (PMC6051575; doi:10.1371/journal.pone.0199295)

## Supporting Information 1

**Figure. Fire salamander secretions SDS-PAGE gel**

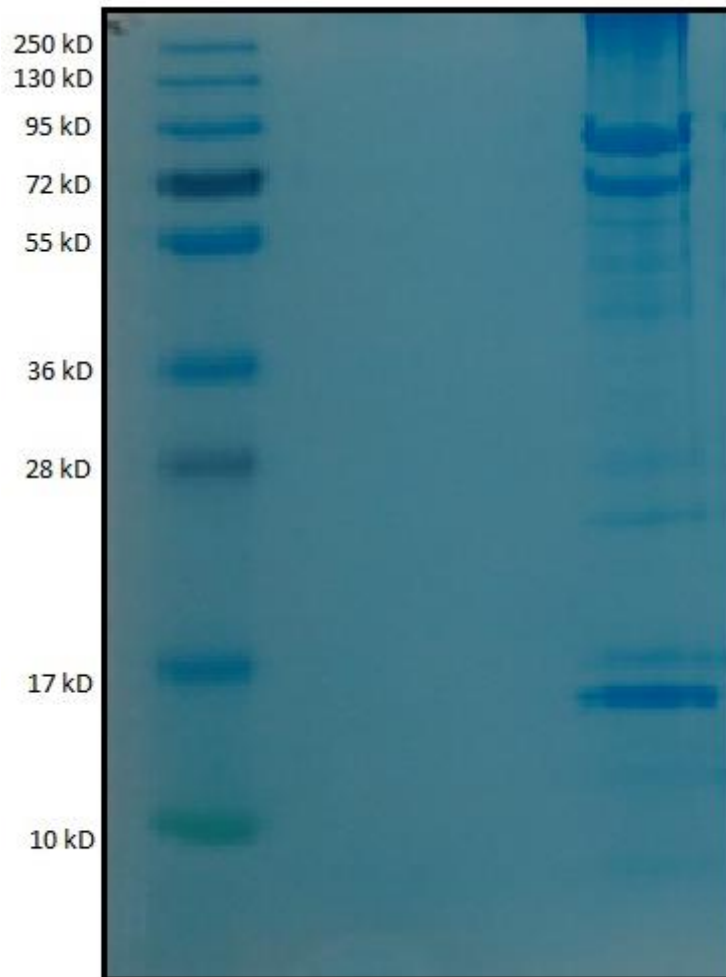

Supplement: S1 Fig — (PDF) [file pone.0199295.s001.pdf]
