# Supplementary material for: Skin mucosome activity as an indicator of Batrachochytrium salamandrivorans susceptibility in salamanders
Source: PLoS One. 2018 Jul 18;13(7):e0199295. doi: 10.1371/journal.pone.0199295 (PMC6051575; doi:10.1371/journal.pone.0199295)
Supplement: S1 Appendix — (PDF) [file pone.0199295.s002.pdf]

Supporting Information 1 Appendix

Mucosome and secretions *Bd* and *Bsal* zoospore assay results

Table A. ***Bsal* zoospore survival (%) versus mucosomes of salamanders and newts.**

Captive fire salamanders	Alpine newt	Fire belly newt	Palmate newt
88.07	42.88	73.23	34.10
90.74	24.96	63.39	24.26
97.73	28.64	58.58	14.65
95.68	14.29	38.90	14.65
64.81	14.29	58.58	19.52
63.58	10.72	38.90	19.52
73.66	35.68		
60.08	7.99		

Wild fire salamanders
81.23
87.68
91.43
87.13
100
70.95
49.88
74.85
62.46
37.43
86.19
68.33
62.46
62.46
45.71

Table B. ***Bsal* zoospore survival (%) in the presence of treated and untreated fire salamander skin secretions and alpine newt mucosomes.** Treatments include; denaturation of proteins with GluC Endoproteinase and removal of small molecules (<50kD) through dialysis.

Fire salamander	Untreated Secretions	Endoproteinase	Dialysis 50kD
	12.03	57.52	12.03
	21.65	81.58	19.17
	2.41	79.32	31.28
**Alpine newt**	**Untreated Mucosome**	**Endoprotienase**	**Dialysis 50kD**
	31.37	93.24	20.71
	37.65	100	51.78
	37.65	72.16	31.07

Table C. Fire salamander secretions and mucosome killing activity versus *Bd* and *Bsal* zoospores.

Secretions		Mucosome	
Bsal	Bd	Bsal	Bd
0	12.50	67.92	29.88
10	20.70	92.12	42.32
0	33.20	92.12	53.94
10	8.32	56.10	44.81
10	0	84.98	60.17
20	0	60.09	44.81
10	0	80.05	43.98
0	0	54.93	43.98
